# Impact of Paleo Diet on Body Composition, Carbohydrate and Fat Metabolism of Professional Handball Players

**DOI:** 10.3390/nu15194155

**Published:** 2023-09-26

**Authors:** Aleksandra Pięta, Barbara Frączek, Magdalena Wiecek, Paulina Mazur-Kurach

**Affiliations:** 1 Department of Sports Medicine and Human Nutrition, Institute of Biomedical Sciences, University School of Physical Education in Krakow, Jana Pawla II 78, 31-571 Krakow, Poland; barbara.fraczek@awf.krakow.pl (B.F.); paulina.mazur@awf.krakow.pl (P.M.-K.); 2 Department of Physiology and Biochemistry, Institute of Biomedical Sciences, University School of Physical Education in Krakow, Jana Pawla II 78, 31-571 Krakow, Poland; magdalena.wiecek@awf.krakow.pl

**Keywords:** Paleo diet, body composition, carbohydrate metabolism, fat metabolism, hormones, physical activity

## Abstract

The Paleo diet (PD) involves a restriction of carbohydrates and increased fat content (35% energy from carbohydrates, 35% energy from fats and 30% energy from protein). The aim of this study was to examine the effect of the PD on body composition, concentration of carbohydrates and lipids, as well as insulin, irisin, adiponectin and leptin in the blood. A total of 25 handball players were assigned to two groups: 14 in the experimental group (PD) and 11 in the control group (CD), using a PD and a rational diet, respectively. Analysis of body mass and body composition (body mass index, fat mass, lean body mass, fat-free mass, muscle mass, bone mineral content and bone mineral density), as well as blood concentration of metabolism markers (glucose, insulin, total cholesterol, HDL-cholesterol, non-HDL-cholesterol, LDL-cholesterol, triglycerides, free fatty acids, β-hydroxybutyrate, irisin, adiponectin and leptin), were determined at the beginning and after 4 and 8 weeks of nutritional intervention. Body mass was lower (*p* < 0.01), and adiponectin blood concentration was higher (*p* = 0.03) in the PD group at the end of the intervention. There were no changes (*p* ≥ 0.05) in body composition and blood levels of other biochemical markers in either group.

## 1. Introduction

The use of a balanced, nutrient-dense diet that covers increased energy, macro- and micronutrient and fluid requirements promotes the maintenance of health potential and optimization of athletic training effects [1,2]. An alternative to the rational diet recommended for athletes is an unconventional diet, defined as a diet characterized by a specific selection of foods and/or the intentional elimination of certain foods/food groups, as well as the introduction of various quantitative modifications in the supply of macronutrients [3]. The variety of diets encourages their use, and emerging evidence on the positive effects of certain dietary strategies, as well as flattering reviews, further motivates people to take up an unconventional way of eating. However, the validity, efficacy and safety of most unconventional dietary strategies (especially over the long term) are controversial. Inappropriate dietary choices and unbalanced diets result in reduced exercise capacity indirectly through negative effects on body composition and health [1,2].

The Paleo diet (PD) involves the restriction of carbohydrates and an increased fat content (35% energy from carbohydrates, 35% energy from fats and 30% energy from protein). Currently, researchers evaluating the nutritional value of the PD classify it as a moderate amount of carbohydrates [4]. Some researchers consider that the Paleo diet is defined by the avoidance of particular food sources rather than a specific macronutrient distribution. The Paleo diet consists of meats, fishes, eggs, vegetables, fruits, roots and nuts; it excludes grains, legumes and dairy products and limits refined sugars, starches, processed foods and oils [5].

The PD has become popular mainly because of its possible health benefits. Positive effects are noticed mainly in obese people (body mass reduction), diabetics (improvement of carbohydrate metabolism), those suffering from cardiovascular disease (normalization of blood pressure) and women with metabolic syndrome (improvement of glucose, insulin sensitivity, reduction of abdominal obesity, normalization of blood pressure and lipid profile) [4,6,7,8,9,10,11]. There have been indications to conclude a positive effect of the PD on other health markers, i.e., reduction of inflammation and normalization of certain hormones (leptin, cortisol) [12,13,14].

Diet and exercise can have different effects on adipose tissue distribution, composition and activity. In light of new discoveries regarding the physiology of adipose tissue, changes in adipokine levels and the signaling connected with them result from qualitative and not quantitative changes in this tissue.

Leptin regulates appetite and food intake, and its biological action is related to its effect on receptors located mainly in the hypothalamus but also in myocytes, hepatocytes, renal cells and endothelial cells. Leptin plays an important role in the regulation of neuroendocrine axes, such as the hypothalamic–pituitary–gonadal axis, the hypothalamic–pituitary–adrenal axis and the hypothalamic–pituitary–thyroid axis [15], and regulates lipid metabolism, contributing to the reduction of lipid concentrations in cells by reducing the synthesis of triglycerides and fatty acids [16]. However, abnormal secretion of the hormone, as well as insensitivity of receptors for the hormone, can cause overweight and obesity and can lead to the development of metabolic syndrome [17]. Excess body fat contributes to dysfunctional leptin release. Abnormal secretion of this hormone leads to insulin resistance by further dysregulating glucose homeostasis and leads to hyperglycemia-related damage [18].

Blood irisin levels were also found to correlate negatively with leptin levels [19] and positively with adiponectin levels [20]. In addition, irisin stimulates glycolysis by increasing lactate synthesis [21]. Irisin has many potential beneficial effects on glucose homeostasis and insulin sensitivity by increasing energy expenditure, enhancing glycogenolysis and decreasing gluconeogenesis, adipogenesis and lipid accumulation [22,23].

The primary adiponectin function is energy homeostasis and is known as “starvation protein” [24,25]. The act of adiponectin is to increase fatty acid oxidation and glucose uptake by cells and increase tissue sensitivity to insulin, which contributes to the maintenance of energy homeostasis [26]. Leptin and adiponectin have opposite roles in energy metabolism [25]. Apart from their metabolic effects, both adipokines are involved in inflammation and immune response, with leptin having proinflammatory and adiponectin having anti-inflammatory properties [27]. The levels of leptin (positively) and adiponectin (negatively) correlate with body mass index (BMI) [28,29].

Adequate availability of energy substrates, regulated hormonally, is of great importance for athletes. For handball players, it is important to efficiently utilize both anaerobic and aerobic energy potential, as this sport is characterized by mixed metabolism [30].

Currently, reduced-carbohydrate dietary strategies are of high interest to athletes [31,32]. The answer to the question of what effects the PD has on endocrine balance in professional athletes remains unclear. Considering the athletes’ objections to the use of the PD, we wanted to assess the impact of an eight-week PD on body composition, carbohydrate and fat metabolism of professional handball players. Prior to the study, the research hypotheses were established: (1) Eight weeks on the Paleo diet does not cause negative changes in lipid profile and carbohydrate metabolism. (2) Eight weeks on the Paleo diet causes a decrease in irisin and leptin and an increase in adiponectin in the blood. (3) Eight weeks on the Paleo diet causes beneficial changes in the body composition of the athletes.

## 2. Materials and Methods

### 2.1. Experimental Design

The research was conducted in accordance with the Declaration of Helsinki, and the research methodology was approved by the Bioethical Committee of the Regional Medical Chamber in Kraków (38/KBL/OIL/2017). The participants were informed in detail about the purpose and course of the study and about the possibility to withdraw from participation in the project at any stage without providing a reason. All the subjects read the written information about the research course, especially nutritional strategy. The participants provided their written consent for voluntary participation in the trial.

Three measurement points were distinguished: baseline—before the start of nutritional intervention or on the first day of the experiment, 4—after four weeks of nutritional intervention, 8—after eight weeks of nutritional intervention.

### 2.2. Dietary Intervention

Based on the determined energy demand and the individual needs for nutrients, body mass and food preferences, the all-day food rations were prepared with the use of the Aliant Dietetic Calculator 4.10.14 (Cambridge Diagnostics, Warsaw, Poland) considering individual assumptions developed by the researcher, considering the isocaloric model of the diet. For a rational diet, the following energy share of macronutrients was adopted: 15–20% of protein, 25–30% of fat and approx. 55–60% of carbohydrates [33,34], and for the PD, accordingly, 20–30% protein, 35–40% fat and 30–35% of carbohydrates [35,36]. The athletes were randomly assigned to two groups differing in the type of diet. Then, on the basis of an analysis of taste preferences, expressed a desire to undertake rational or paleo diet. Eight weekly food rations (784 for the subjects in the Paleo diet and 616 in the rational diet group) were created for each athlete and then prepared by the catering company. During the experiment, the athletes did not take any supplements influencing the resting and exercise metabolism.

### 2.3. Characteristics of Participants and Measurement Methods

The research group consisted of 25 male athletes training in handball: 14 in the experimental group (the Paleo diet (PD)) and 11 in the control group (rational diet (control diet, CD)). Handball players participating in the study were aged 18–35 and members of one team—the 2nd league team from Poland. The research was undertaken during the preparatory period. Players received an average of 6 discipline-specific training units per week, lasting an average of 2 h/day. Training loads were the same for everyone. On the first day of the study, on an empty stomach, and the next day, on an empty stomach after 4 and 8 weeks of nutritional intervention (PD or CD), a qualified nurse took blood samples to determine biochemical blood indicators: glucose, insulin, total cholesterol (TC), HDL-cholesterol (HDL-C), non-HDL-cholesterol (non-HDL-C), LDL-cholesterol (LDL-C), triglycerides (TG), free fatty acids (FFA), β hydroxybutyrate (β-HB), irisin, leptin and adiponectin. The subjects’ body heights were measured using an anthropometer to the nearest 0.5 cm. The body composition was analyzed at the same measurement points using dual-energy X-ray absorptiometry (DXA) using the Lunar iDXA^TM^ instrument. Body mass (BM), body mass index (BMI), fat mass (FM) (% and kg), fat-free mass (FFM) (% and kg), lean body mass (LBM)—body mass without adipose tissue and bone minerals (% and kg), bone mineral content (BMC) (g) and bone mineral density (BMD) (g/cm^2^) were analyzed. The content of muscle mass (MM) (% and kg) was calculated using the Kim formula [37].

Five milliliters of blood was collected into tubes (Becton Dickinson, Franklin Lakes, NJ, USA) containing an anticoagulant (K2EDTA—dipotassium ethylenediaminetetra-ace-tate dihydrate) and a protease inhibitor (aprotinin 0.6 TIU/1 mL of blood) as well as with a clotting activator. Immediately after collection, the blood was mixed by inverting the tube several times, avoiding shaking and then immediately centrifuged for 15 min at 4 °C, RCF 1000× *g* (MPW-351R, Med. Instruments, Warsaw, Poland). The concentration of irisin (µg/mL), leptin (ng/mL) and adiponectin (µg/mL) in the blood plasma was determined by enzyme immunoassay (ELISA) method according to the manufacturer’s specification, using reagent kits from BioVendor (Karasek, Czech Republic), Human Irisin ELISA Kit RAG018R, respectively, detection range 0.001–5 μg/mL, intra-assay CV < 8.2%, inter-assay CV < 9.7%, Human Leptin ELISA Kit RD191001100, detection range 0–50 ng/mL, intra-assay CV < 7.6%, inter-assay CV < 6.7%, Human Adiponectin ELISA Kit RD191023100, detection range 2–150 ng/mL, intra-assay CV < 4.4%, inter-assay CV < 6.6%. The results were read from a standard curve made during each of the determinations (Infinite M200 PRO TECAN, Grödig, Austria).

Plasma glucose concentration (mmol/L) was determined by hexokinase method on COBAS apparatus (Roche Diagnostic, Rotkreuz, Switzerland).

Serum insulin concentration (µIU/mL) was determined with Roche’s Elecsys Insulin test by the ECLIA electroimmunochemical method on a Cobas instrument.

Free fatty acid concentration (mmol/L) was determined in serum by colorimetric method on an RX Monza analyzer (Ranbut, Randox, Crumlin, UK). Beta-hydroxybutyrate concentration (mmol/L) was determined on an RX Monza analyzer by a kinetic enzymatic method for the determination of D-3-hydroxybutyrate in serum. The method is based on the oxidation of D-3-hydroxybutyrate to acetylacetate by the enzyme 3-hydroxybutyrate dehydrogenase.

The lipid profile was determined in the serum. TC (mmol/L) was determined by enzymatic, colorimetric, CHOD-PAP method on COBAS apparatus (Roche Diagnostic, Switzerland). HDL-C (mmol/L) was determined by homogeneous colorimetric, enzymatic method on COBAS apparatus, non-HDL-C (mmol/L) was calculated. Triglycerides (mmol/l) were determined by enzymatic, colorimetric, GPO-PAP method on COBAS apparatus. LDL-C (mmol/L) was calculated from the Friedwald formula.

### 2.4. Statistical Analysis

The PQStat statistical package, version 1.8.0.338, was used for the statistical analysis of the collected results. The basic characteristics of the examined variables were calculated, i.e., mean and standard deviation (SD) or median (Me), upper quartile (Q1), lower quartile (Q3). Excluding hormones, the distribution of data was different than normal (W Shapiro–Wilk Test), so the results of the analyzed scales between the groups PD and CD were compared with the non-parametric Mann–Whitney U-Test. Differences in results between weeks 4 and baseline and weeks 8 and baseline in the PD group and in the CD group were determined using the Friedman test, followed by Dunn’s post-hoc test with Bonferroni correction, and the trend of change was estimated using the Page test. The results of hormone levels were analyzed by a two-factor analysis of variance in which the grouping factor was the division into the PD and CD groups; the second factor was the measurement repeated over time in successive stages. Post-hoc analyses were conducted using Duncan’s test.

## 3. Results

Comparing the groups eating the PD and the CD, no differences (*p* ≥ 0.05) were found in the distributions of basic data from age and anthropometric measurements: PD: 21.00 (20.00; 23.00) years and CD: 23.00 (22.00; 25.00) years, as well as in anthropometric parameters: body height, respectively: 1.88 (1.80; 1.94) m and 1.84 (1.80; 1.89) m, body mass: 90.18 (85.40; 100.00) kg and 87.1 (83.90; 95.50) kg, BMI: 25.92 (25.43; 27.15) kg/m^2^ and 25.90 (25.68; 27.43) kg/m^2^, fat content: 22.35 (16.60; 28.90)% and 18.60 (15.80; 23.20)%, muscle content: 42.25 (39.00; 45.20)% and 43.50 (42.80; 45.90)%.

As a result of the applied nutritional interventions, significant changes were found only in the PD group. Body mass was significantly lower (*p* < 0.01), and adiponectin concentration was significantly higher (*p* = 0.03) in the PD at the end of the intervention. There were no differences (*p* ≥ 0.05) in body mass and body composition (BMI, FM, LBM, FFM, MM, BMC and BMD) (Table 1), glucose, insulin, FFA, β-HB, TC, LDL-C, HDL-C, non-HDL-C, TG as well as adiponectin, irisin and leptin concentrations between PD and CD groups in each series during the dietary intervention (Table 2). In both groups, concentrations of all assessed indices of carbohydrate and fat metabolism remained within reference values throughout the experiment [38] (Table 2).

## 4. Discussion

We obtained results demonstrating the beneficial effect of the Paleo diet, which was shown by a decrease in body mass, although without changes in body composition, and by an increase in blood adiponectin concentrations in handball players in this dietary intervention group.

Our last meta-analysis [4] considered 21 studies analyzing the effects of the PD in groups of healthy inactive and unhealthy people on health status. Overall, the PD and various healthy diets (e.g., the Mediterranean diet, diet in accordance with Nordic Nutrition Recommendation, the Dutch Health Council, the American Diabetes Association and the American Heart Association) caused decreases in TC, LDL-C and TG, albeit the impact of the PD was stronger. Among long-term (over 6 months) studies, only the PD caused a decrease in TC and LDL-C. What is more, PD caused a statistically significant decrease in glucose, insulin, Homeostatic Model Assessment—Insulin Resistance (HOMA-IR) and glycated hemoglobin (HbA1c) in the short term (up to 6 months)—contrary to various CDs, which caused only a significant decrease in HbA1c. In the long term, the PD caused no changes in any carbohydrate metabolism indicators [4,6,7,8,9,10,11]. The results of our study, an 8-week intervention with the PD in a group of handball players, partly coincides with the results of the meta-analysis. In our research, the eight-week application of PD had a partially positive effect on body composition through a decrease in BM. The effect on BMI, FM (% and kg), FFM, LBM and MM were inconclusive in PD. There were no differences in carbohydrate (glucose, insulin) and fat (FFA and β-HB, TC, LDL-C, HDL-C, non-HDL-C, TG levels) metabolism concentrations during the dietary intervention. In regard to adiponectin in our research, it increased throughout the experiment, and after 8 weeks, its level was higher in the group receiving the PD. There were no changes in irisin and leptin concentrations. Eight-week intervention of the normoenergetic PD resulted in a decrease in body mass, which may be explained by changes in the diet composition and next adiponectin concentration.

However, there have been other indications that the PD has positive effects, i.e., it reduces inflammation and normalizes certain hormones (leptin, cortisol). The data show that the PD, in combination with physical activity, is effective in improving body composition and metabolic balance, including insulin sensitivity, control of glycemia and leptin control in Type 2 Diabetes (T2D), and might positively affect exercise capacity [12,13,14]. In addition, genetic and in vitro studies indicate an insufficient adaptation of leptin receptors to a cereal grain-based diet [35]. Therefore, cereals can hypothetically lead to leptin resistance and, thus, higher leptin concentrations. Studies show lower leptin levels as a result of using a PD that is practically devoid of cereals compared to a diet such as a diabetic diet, which is based on whole grain products. This supports this view and might be the mechanism behind the improvement of blood glucose and lipid control and the greater feeling of satiety associated with the Paleolithic diet [39], which does not quite connect with the results of our own study because there were no differences in the context of leptin concentrations (insignificant decrease). The other study demonstrated that after 8 weeks of chronic exercise training, leptin, irisin and insulin levels decreased [40]. Moreover, longer durations of exercise (more than 60 min), associated with increased energy release, can reduce leptin concentrations [41]. In our study, we obtained improvements in BM and adiponectin levels after applying the PD for 8 weeks. In de Luis et al. [42], a high-protein/low-carbohydrate hypocaloric diet showed a higher weight loss, and insulin and HOMA-IR decreased after 9 months, compared to a standard hypocaloric diet. The improvement in adipokine levels was similar with both diets. In addition, in both diets, leptin levels decreased [42]. According to several studies, different diets and nutritional supplementations can modify leptin concentration, while the impact of physical activity and weight reduction appear to be key [43,44]. Similarly, as far as insignificant changes in irisin concentrations are concerned, they seem to be dependent on physical activity rather than on a specific diet. Acute exercise can increase the concentration of circulating irisin, and chronic exercise can improve the irisin metabolic dynamic and selectively increase the circulating irisin concentration of subjects [45], which was not the case for handball players who engage in daily physical activity. Anastasilakis et al. [45] showed that the irisin levels were not affected by intake of a standardized meal and were not associated with caloric intake or diet quality. Thus, in healthy, young individuals, circulating irisin displays a day–night rhythm, is correlated with lean body mass and increases acutely after exercise [45], which may connect with our findings. What is more, Shirvani et al. [46] observed that primarily aerobic exercise and not caloric restriction diet influences irisin expression. Interestingly, it has also been shown that 3-n PUFA supplementation (a dosage of 1250 mg three times per day) increases irisin expression in diabetes [47]. Moreover, the nutraceuticals that have been most studied as modulators of irisin expression are polyphenols [25], which, as well as omega-3 PUFAs, are abundant in the PD, which showed no effect in our study.

Changes in the concentration of adipocytokines are inter-related [43]. In agreement with the above discussion about nutritional interventions and leptin levels, antioxidant compounds of PD may be a promising natural coadjuvant in the treatment of obesity and cardiovascular disease, acting on adiponectin levels. The results in animal models demonstrate that the consumption of hyperlipidemic diets rich in SFA reduces the levels of adiponectin, while the diets rich in PUFA and supplementation with omega-3 increase gene expression and plasma levels [48]. In our study, the use of the PD increased the concentration of adiponectin in the PD group, even with a higher content of saturated fatty acids (SFA) and cholesterol in the PD (3 and 2 times higher than the norm, respectively) [49]. Importantly, beneficial features of PD that might be responsible for these effects are low energy density, high fiber, polyphenol, and PUFA content.

A positive effect of the other, similar alternative nutritional strategy—ketogenic diet (KD)—on the lipid profile in athletes was also observed, which, according to the authors, could be due to the predominance of PUFA in the diet used [31]. Other scientific evidence suggests that the use of low-carbohydrate diets adversely affects the lipid profile and promotes atherosclerotic changes in athletes [50]. A higher intake of SFA and cholesterol and lower fiber intake may be the cause of hypercholesterolemia, as shown in studies of 20 ultra-endurance athletes [51]. A low carbohydrate diet (LCD) has also been shown to be associated with higher mortality and metabolic disorders if it contains large amounts of foods of animal origin, especially saturated fats [52]. Our research showed that the 8-week PD had no effect on the lipid profile. The reason for the lack of adverse changes in the lipid profile could be the high content of PUFA (nuts, seeds, oils) and dietary fiber in the diet (because of the high content of fruits and vegetables in PD). On the other hand, it should not be forgotten that the PD provided significantly more proinflammatory SFA and cholesterol. This lipid profile suggests that the PD is not a threat to stimulate cardiovascular disease resulting from lipid profile disorders. Therefore, it can be concluded that with a high supply of fiber and PUFA, even the high content of saturated fatty acids and cholesterol in the diet did not cause a negative effect on the lipid profile. This dietary procedure is recommended to representatives of team sports in order to reduce fat mass, lipid profile disorders and insulin resistance [49].

Scientists dealing with the topic of alternative strategies list several possible positive consequences of undertaking various nutritional strategies, i.e., training with different amounts of carbohydrate availability [53]. The aim of the experiments undertaken by physiologists and nutritionists is to assess the relationship between the type of diet and the use of energy substrates during efforts of different intensities. Mechanisms of the influence of diet on exercise metabolism remain ambiguous. At the beginning of the second decade of the 21st century, it was confirmed that a low-carbohydrate diet increases fat metabolism and “saves” glycogen reserves [54,55], and the availability of carbohydrates and lipids largely determines the nature of exercise metabolism. With a high availability of free fatty acids (FFA), the body prefers to use them, saving much poorer carbohydrate resources [31,54,56,57,58,59,60,61]. Manipulating glycogen resources is now recognized as an important tool in optimizing training adaptations [53]. Increasing the body’s ability to use ketone bodies (KB) as an energy source generally occurs with two types of manipulation: (1) by restricting dietary carbohydrate intake for prolonged periods, the body metabolically adapts to using KB instead of glucose, keto-adaptation [54]; (2) by supplementing with ketone esters, wherein fuel consumption instantly shifts from carbs to ketones [55,60,62,63]. In our research, no changes in carbohydrate and fat metabolism were found as a result of intervention with a diet with a reduced supply of carbohydrates compared to the control group. Studies analyzing the effect of the KD on carbohydrate metabolism have shown improvements in fasting glucose and insulin levels among athletes [64,65]. Other studies with limited carbohydrate intake have shown a decrease in blood insulin levels at rest or during exercise [32,64,66]; unchanged concentrations of this hormone were observed [31,54,56]. Research shows that lipolysis and fat oxidation increased as insulin levels decreased [64,65]. Elevated ketones are necessary to trigger decreased insulin production and thus increase fat utilization [67]. Perhaps that is why, in our research, no decrease in blood insulin was observed.

It is worth noting that our research analyzed the impact of the PD in professional athletes. Physical activity modifies the expression not only of myokines but also of other molecules, such as adiponectin [25,43]. However, it is questionable whether the positive impact of the PD on health is caused by the elimination of specific products from the diet, e.g., foods rich in some anti-nutrients, high content of omega-3, prebiotic (high amount of fiber) and polyphenols or even a reduction in body weight. It has been known for a long time that the correct structure of the body directly affects the state of health. Exercise could be a confounding element in evaluating the effectiveness of nutritional interventions. Considering the importance of physical activity, it could be appropriate to always test a specific nutritional strategy in association with exercise and not consider the two aspects individually [25].

We recognize several limitations in our study. One major drawback of this study is the small sample size, making it unable to investigate possible significant associations between the effects of the Paleo diet and markers of carbohydrate and lipid metabolism. What is more, limitations in the selection of subjects were the high sports level of the handball players, the long study period (which is also the main advantage of the study) and the specific products available in the Paleo diet (which did not always meet the food preferences of the handball players). On the other hand, preparing meals for each athlete with the assumption of maintaining balanced energy, especially with high energy demand and the specific composition of the Paleo diet, eliminated the possibility of methodological errors.

## 5. Conclusions

Our data indicate that the introduction of the Paleo diet has a positive effect on body mass and adiponectin concentration in the serum of handball players. It is still puzzling to what extent the hormone levels were affected by the diet or body mass reduction and/or physical activity. Further research is required on the influence of nutritional intervention with physical efforts of varying intensity on the secretion of adipocytokines/myokines.

## Figures and Tables

**Table 1:** Body composition at different measurement points during both diets.

Indicator	Measurement Point	PD	CD
		Me (Q1;Q3)	Me (Q1;Q3)
BM (kg)	baseline	90.18 (86.40; 100.00)	87.10 (83.90; 95.50)
	4	90.12 (78.80; 99.10)	86.30 (82.20; 92.20)
	8	88.45 (78.00; 95.80) **	85.00 (82,30; 93.40)
BMI (kg/m^2^)	baseline	25.92 (25.43; 27.15)	25.90 (25.68; 27.43)
	4	24.76 (23.76; 26.41)	25.17 (24.34; 27.25)
	8	24.97 (24.37; 26.44)	25.37 (24.54; 27.53)
FM (kg)	baseline	18.70 (13.90; 25.70)	16.30 (12.70; 19.30)
	4	16.50 (12.20; 20.90)	14.30 (10.80; 17.30)
	8	14.70 (11.90; 19.00)	13.60 (9.90; 15.80)
FM (%)	baseline	22.35 (16.60; 28.90)	18.60 (15.80; 23.20)
	4	19.45 (15.00; 27.30)	17.00 (13.40; 21.30)
	8	17.30 (13.70; 21.90)	17.20 (12.60; 20.00)
LBM (kg)	baseline	69.30 (64.02; 72.20)	67.50 (63.80; 73.90)
	4	68.70 (63.50; 70.90)	67.70 (62.30; 74.00)
	8	68.30 (64.10; 71.60)	68.70 (63.80; 73.90)
LBM (%)	baseline	75.25 (73.40; 81.30)	76.90 (70.90; 80.40)
	4	77.10 (73.90; 81.80)	79.50 (73.30; 82,.80)
	8	78.50 (74.80; 82.50)	79.20 (73.80; 83.40)
FFM (kg)	baseline	71.90 (67.50; 75.10)	71.20 (63.80; 78.50)
	4	70.60 (67.10; 73.40)	71.50 (63.70; 78.00)
	8	71.30 (67.80; 72.00)	72.40 (64.70; 80.40)
FFM (%)	baseline	78.75 (72.20; 81.90)	82.20 (75.30; 84.90)
	4	81.15 (73.80; 84.90)	83.80 (78.10; 87.30)
	8	82.00 (78.60; 84.50)	83.60 (78.20; 88.00)
MM (kg)	baseline	38.75 (35.10; 41.10)	37.80 (36.50; 43.50)
	4	37.00 (33.80; 40.30)	38.10 (34.10; 42.70)
	8	37.50 (35.40; 41.00)	38.30 (34.20; 42.40)
MM (%)	baseline	42.25 (39.00; 45.20)	43.50 (42.80; 45.90)
	4	41.45 (38.70; 45.10)	44.20 (41.50; 46.30)
	8	43.00 (41.40; 45.80)	44.70 (41.50; 45.60)
BMC (g)	baseline	3.77 (3.54; 4.18)	3.78 (3.65; 4.35)
	4	3.76 (3.51; 4.19)	3.81 (3.41; 4.27)
	8	3.71 (3.51; 4.14)	3.83 (3.42; 4.24)
BMD (g/cm^2^)	baseline	1.41 (1.35; 1.58)	1.47 (1.39; 1.62)
	4	1.44 (1.34; 1.51)	1.45 (1.40; 1.52)
	8	1.43 (1.36; 1.50)	1.47 (1.36; 1.55)

Abbreviations: PD—Paleo diet, CD—control diet, Me—median, Q1—upper quartile, Q3—lower quartile, BM—body mass, BMI—body mass index, FM—fat mass, LBM—lean body mass, FFM—fat-free mass, MM—muscle mass, BMC—bone mineral content, BMD—bone mineral density, **—highly significant intragroup differences compared with the baseline at the level of *p* < 0.01.

**Table 2:** Concentration of selected metabolic indicators at different measurement points for both diets.

Indicator	Measurement Point	PD	CD
		Me (Q1; Q3)	Me (Q1; Q3)
Glucose (mmol/L)	baseline	5.05 (4.83; 5.40)	5.06 (4.94; 5.21)
	4	4.96 (4.70; 5.18)	4.83 (4.58; 5.15)
	8	4.99 (4.60; 5.33)	4.96 (4.77; 5.05)
Insulin (µIU/mL)	baseline	7.13 (6.13; 8.75)	7.25 (5.37; 10.60)
	4	6.60 (4.80; 9.72)	6.76 (5.95; 7.90)
	8	6.32 (5.50; 8.48)	5.54 (4.72; 7.39)
FFA (mmol/L)	baseline	0.52 (0.25; 0.68)	0.24 (0.15; 0.41)
	4	0.35 (0.28; 0.57)	0.52 (0.22; 0.76)
	8	0.29 (0.21; 0.54)	0.39 (0.19; 0.64)
β-HB (mmol/L)	baseline	0.05 (0.04; 0.07)	0.05 (0.04; 0.15)
	4	0.10 (0.05; 0.17)	0.08 (0.05; 0.21)
	8	0.11 (0.04; 0.18)	0.08 (0.05; 0.12)
TC (mmol/L)	baseline	3.83 (3.58; 4,31)	3.86 (3.52; 4.75)
	4	4.35 (3.42; 4.65)	3.81 (3.49; 4.83)
	8	4.26 (3.91; 4.72)	3.93 (3.66; 4.50)
HDL-C (mmol/L)	baseline	1.39 (1.22; 1.91)	1.44 (1.22; 1.78)
	4	1.39 (1.18; 1.69)	1.40 (1.09; 1.62)
	8	1.53 (1.36; 1.86)	1.37 (1.11; 1.78)
non-HDL-C (mmol/L)	baseline	2.53 (2.08; 2.99)	2.44 (1.95; 3.10)
	4	2.75 (2.08; 3.21)	2.25 (1.99; 3.28)
	8	2.78 (2.65; 3.10)	2.60 (1.85; 3.13)
LDL-C (mmol/L)	baseline	2.06 (1.71; 2.52)	1.84 (1.56; 2.42)
	4	2.47 (1.80; 2.86)	1.89 (1.67; 2.92)
	8	2.26 (1.76; 2.67)	2.08 (1.53; 2.71)
TG (mmol/dL)	baseline	1.07 (0.89; 1.67)	0.80 (0.70; 1.30)
	4	0.76 (0.61; 1.05)	0.81 (0.69; 1.09)
	8	0.97 (0.79; 1.15)	1.00 (0.68; 1.54)
		Mean ± SD	Mean ± SD
Adiponectin (µg/mL)	baseline	4.54 ± 2.97	6.99 ± 5,61
	4	4.62 ± 3.39	6.01 ± 4.30
	8	5.14 ± 3.42 *	6.35 ± 4.74
Leptin (ng/mL)	baseline	3.10 ±1.75	5.09 ± 5.27
	4	1.86 ± 1.63	3.23 ± 3.78
	8	2.41 ± 2.25	2.78 ± 3.16
Irisin (µg/mL)	baseline	8.85 ± 3.36	9.16 ± 2.74
	4	8.11 ± 3.24	8.04 ± 3.47
	8	6.88 ± 2.64	6.59 ± 3.14

Abbreviations: PD—Paleo diet, CD—control diet, Me—median, Q1—upper quartile, Q3—lower quartile, SD—standard deviation, FFA—free fatty acids, β-HB-beta hydroxybutyrate, TC—total cholesterol, HDL-C—high-density lipoprotein cholesterol, non-HDL-C—non-high-density lipoprotein cholesterol, LDL-C—low-density lipoprotein cholesterol, TG—triglycerides, *—statistically significant intragroup differences compared with the baseline at the level *p* < 0.05.

## Data Availability

Not applicable.
